# Assessment of Fall Risk in Neurological Disorders and Technology: Relationship Between Silver Index and Gait Analysis

**DOI:** 10.3390/s26030840

**Published:** 2026-01-27

**Authors:** Letizia Castelli, Chiara Iacovelli, Anna Maria Malizia, Claudia Loreti, Lorenzo Biscotti, Pietro Caliandro, Anna Rita Bentivoglio, Paolo Calabresi, Silvia Giovannini

**Affiliations:** 1 Department of Neurosciences, Università Cattolica del Sacro Cuore, 00168 Rome, Italy; 2 Department of Emergency, Anaesthesiology and Intensive Care Medicine, Fondazione Policlinico Universitario A. Gemelli IRCCS, 00168 Rome, Italy; 3 UOSD Riabilitazione Multidimensionale e Tecnologie Integrate, Fondazione Policlinico Universitario A. Gemelli IRCCS, 00168 Rome, Italy; 4 Unità Supporto Amministrativo Dipartimenti Universitari, Università Cattolica del Sacro Cuore, 00168 Rome, Italy; 5 UOC Neurologia, Fondazione Policlinico Universitario A. Gemelli IRCCS, 00168 Rome, Italy; 6 Department of Geriatrics and Orthopaedics, Università Cattolica del Sacro Cuore, 00168 Rome, Italy

**Keywords:** neurological disease, falls, balance, gait analysis, innovative technologies

## Abstract

Falls are one of the most common and devastating effects of neurological diseases, especially in patients with stroke outcomes, Parkinson’s Disease (PD), and Multiple Sclerosis (MS). To prevent negative outcomes and guide tailored rehabilitation, it is necessary to identify risk factors early. The current study aims to assess whether and how the risk of falling is related to spatiotemporal and kinematic parameters in stroke, PD, and MS. It also seeks to determine how these factors can help manage patients and identify more personalized and appropriate rehabilitation treatments. Ninety patients with neurological disorders (stroke, PD, and MS) underwent eight weeks of home-based rehabilitation using the ARC Intellicare device or following a paper-based protocol. At baseline (T0) and at the end of the protocol (T2), they were assessed using the Silver Index of the hunova^®^ robotic platform to evaluate fall risk, and instrumental gait analysis to record spatiotemporal and kinematic parameters of walking. Statistical analysis showed moderate and significant correlations between the Silver Index and gait spatiotemporal parameters such as stance and swing phase, both in affected (T0, *p* = 0.007; T2, *p* = 0.017) and unaffected side (T0, *p* = 0.022; T2, *p* = 0.008), double support in affected side (T0, *p* = 0.002; T2, *p* = 0.005), cycle length in affected (T0, *p* = 0.007; T2, *p* = 0.003) and unaffected side (T0, *p* = 0.008; T2, *p* = 0.003), and cadence (T0, *p* = 0.025; T2, *p* = 0.003) in stroke patients. No significant results emerged in the PD and MS. No population showed significant correlations between the Silver Index and gait kinematic parameters. The Silver Index may reflect distinct patterns of instability in post-stroke gait, but in PD and MS, multiple factors influence the risk of falling that instrumental gait analysis cannot fully capture, requiring a more extensive and multidimensional approach that includes cognitive aspects.

## 1. Introduction

Falls are one of the most common and clinically relevant complications in populations affected by neurological disorders. In gait and balance disorders typical of stroke, Parkinson’s Disease (PD), and Multiple Sclerosis (MS), the frequency of falls is significantly higher than in the general population and often occurs repeatedly, with serious consequences impacting safety and quality of life. Falls occur in about 34% of neurological patients within a year, and gait abnormalities are the main cause of falls in 55% of cases [1]. Beghi et al. [2] have shown that patients with stroke, PD, or MS fall at least once, and a significant proportion of these events are subject to recurrent falls or fall-related injuries [2]. Stroke is associated with a high rate of falls in the first few months after the event; according to a comprehensive review, up to 73% of survivors fall in the first year after the acute event [3]. Similarly, in patients with PD, the annual frequency of falls can vary between 45% and 68%, which is much higher than that observed in healthy peers [4]. In MS, a prevalence of fallers ranging from 30% to 63% has been identified over a period of 1 to 12 months [5].

Several pieces of evidence emphasize that an integrated assessment of walking is essential to understand the mechanisms of risk and guide targeted preventive strategies [6]. The link between gait and falls is central to neurological disorders: biomechanical, cognitive, and sensory alterations emerge as key risk modifiers. These relationships have been documented in reviews and meta-analyses and in prospective studies correlating kinematic and spatiotemporal gait parameters with fall events [7,8].

From a pathophysiological point of view, the mechanisms linking gait to the risk of falling are multifactorial. They include spatiotemporal alterations (walking speed, stride length, cadence), stride variability and asymmetry, dynamic instability (reduced local stability), as well as sensory components (proprioception, visuo-vestibular integration), motor (strength, tone, muscle timing), and cognitive (attention, executive function, dual-tasking) components. In each of the three neurological disorders considered, these elements combine in specific ways: for example, asymmetry and impaired dynamic stability are common features in stroke survivors; the tendency toward reduced speed and short stride length is frequently reported as being associated with an increased risk of falling in PD; stride variability and reduced adaptability are frequently observed in MS [7,8].

Stroke survivors often exhibit gait asymmetries, particularly pronounced in the affected limb, such as shorter step lengths and increased step width, which are significant predictors of tripping and falling [9,10]. Dynamic balance measures, such as the Timed Up and Go (TUG) test, and reduced mediolateral pelvic displacement during walking are strong predictors of falls [11], as are the altered gait velocity and the cycle time in these patients; decreased velocity and prolonged cycle time are associated with near falls [12].

In PD patients, gait abnormalities—such as reduced speed, short step length, irregular cadence, and freezing of gait—are among the most consistent predictors of falls [4]. Non-motor comorbidities in PD, such as autonomic symptoms, anxiety, fatigue, and cognitive dysfunction, show significant correlations with fall risk [13]. These findings suggest that unstable gait is not only a matter of motor deficits but is also modulated by executive functions, attention control, and behavioral adaptation. Moreover, interventions aimed at improving specific components of gait patterns may play a role in mitigating fall risk, but there remains little direct evidence on the long-term effect of such biomechanical modifications on actual fall outcomes [7]. Wearable sensors have been used to monitor these parameters, providing continuous assessment of fall risk in real-world settings [14].

In MS patients, the use of walking aids, longer disease duration, and progressive form are among the factors most strongly associated with falls [5,15]. In addition, stride variability (stride-to-stride variability), reduced postural stability [15], and gait speed are critical factors influencing fall risk. Moreover, MS patients show limitations in dynamic balance, as evidenced by performance on the TUG test and Dynamic Gait Index [16]. Backward walking velocity and step length serve as significant indicators of fall risk in MS patients [17].

Gait analysis has become a sensitive diagnostic and understanding tool for the interplay between neurological disorders and gait patterns, revealing important information about a patient’s conditions and rehabilitation [18]. Recently, Bove et al. [19] used gait analysis to assess walking before and after a home trial with the ARC Intellicare device (Henesis Srl, Parma, Italy), emphasizing that sensor-guided telerehabilitation appears to be particularly effective in remodeling complex motor patterns such as walking after an acute event such as a stroke. Regarding the risk of falls in neurological disorders, a recent study [20] demonstrated that the Silver Index algorithm [21,22], performed by the hunova^®^ robotic platform (Movendo Technology srl, Genoa, Italy), is an effective tool for assessing that risk in neurological disorders. The impact of specific spatiotemporal gait parameters on the risk of falling is well known in the literature, but this second variable is often not objectively quantifiable. Using the Silver Index to assess the risk of falling makes this variable quantifiable and measurable, providing more accurate information and enabling statistical inference and correlation with other parameters.

The aim of the present study is to evaluate whether and how the risk of falling (assessed with the hunova^®^ robotic platform) and the spatiotemporal and kinematic parameters (recorded using instrumental gait analysis) are associated in stroke, PD, and MS, and how they can contribute to patients’ management and to the identification of a more appropriate rehabilitation treatment. Our research hypothesis is that the presence of significant associations between the Silver Index and gait parameters is associated with fall risk, enabling the detection of early markers of instability, allowing the definition of tailored rehabilitation treatment, and the monitoring of recovery in stroke, PD, and MS. On the other hand, the absence of a significant association could indicate the limitations of gait-only assessments in one or more diseases, suggesting the need for a more personalized and disease-specific evaluation strategy.

## 2. Materials and Methods

This is an ancillary study to the one recently published by Bove et al. [19], whose objective was to evaluate the feasibility of home telerehabilitation using the ARC Intellicare device in a population of patients with neurological disorders. According to the inclusion criteria of the main study [19], a total of 90 patients who met the following inclusion criteria were included in the study. To be included in the study, patients had to possess the following characteristics: stroke outcomes, occurred within the last 12 months, age between 50 and 75 years, and modified Rankin Scale score between 2 and 3 [23]; diagnosis of PD (Movement Disorders Society criteria [24]), age between 50 and 75 years, clinical stage between 1 and 3 according to Hoehn and Yahr [25], and stable antiparkinsonian therapy for at least 30 days; or diagnosis of MS (McDonald 2017 criteria [26]), age between 18 and 60 years, and Expanded Disability Status Scale score between 3.5 and 6.5 [27]. On the other hand, patients with a Mini Mental State Examination score < 24 [28], corticosteroid therapy or initiation of a new disease-modifying drug for MS in the three months prior to enrolment, seizures or recent history of dizziness and/or falls, Tinetti Balance Scale score < 19 [29], active and/or unstable comorbidities, symptomatic orthostatic hypotension, and severe orthopedic comorbidities were excluded. Paragraph 2.1 specifies the distribution of patients for each condition considered.

According to the methodology of the main study [19], patients were randomized in a 1:1 ratio to the experimental group, which underwent home telerehabilitation with the ARC Intellicare device, and the control group, which underwent home rehabilitation according to a paper-based protocol provided by an expert physical therapist. All patients, regardless of randomization group, underwent home-based rehabilitation treatment for 60 min per day, 3 days per week for 8 weeks, and were assessed at the beginning of the study (baseline, T0), at the end of the 8-week treatment period (T2), and after 4 weeks of observation (follow-up, T3).

ARC Intellicare is a device that combines the use of wearable sensors, a mobile device, and artificial intelligence algorithms [30]. The home configuration of the device allows video calls to be made with the physiotherapist in case of need or for scheduled monitoring, as well as viewing video tutorials of the exercises, to guide patients in the unsupervised performance of the proposed rehabilitation activities. The ARC Intellicare device contains a library of exercises for mobility coordination, balance, and muscle strengthening already used in clinical practice [31].

Patients in the ARC Intellicare group and those in the paper protocol group performed the same exercises, despite the different methods of administration. The progression of the exercises has already been detailed in the previous article [20].

The study by Bove et al. [19] evaluated the efficacy and safety of ARC Intellicare in several neurological disorders, such as stroke, PD, and MS, while this study examined information on gait and fall risk in participating patients at baseline (T0) and at the end of 8 weeks of treatment (T2).

Instrumental gait analysis was performed using a Smart D500 optoelectronic stereophotogrammetric system (BTS S.p.A., Garbagnate Milanese, MI, Italy). The system consists of eight cameras, two force platforms, a traditional two-camera video recording system, and a system for synchronizing and processing the various signals. The Davis protocol with 22 markers was used to identify anatomical landmarks [32]. All spatiotemporal parameters of the gait cycle (stance phase, swing phase, double support phase, gait cycle time, cadence, step length, mean velocity, gait cycle length, and step width) and kinematic parameters (range of motion) of the hip, knee, and ankle joints were calculated (Table S1). Fall risk analysis, as detailed in a recent article [20], was performed using the hunova^®^ robotic platform, specifically the Silver Index algorithm [20,21,22]. Instrumental gait analysis and fall risk assessment using hunova^®^ [33] were performed by physiotherapists assigned to the patient assessment, who were blinded to the randomization group.

This study was conducted in accordance with specific national laws and ethical standards outlined in the 1964 Declaration of Helsinki and its subsequent amendments. The Institutional Ethics Committee approved the study protocol (Prot. 0014656/23, NCT06032468).

### Statistical Analysis

Considering that the main study was designed as a feasibility study [19], no formal estimate of the sample size was made. However, based on Julious’s rules [34], 90 subjects (30 for stroke, 30 for PD, and 30 for MS) were included in the study and randomized into two groups (ARC Intellicare group, the experimental group, and paper-based protocol group, the control group).

The sample was described in its clinical and demographic variables. Quantitative variables were summarized with mean and standard deviation (SD). The Shapiro–Wilk probability test was used to assess the normality of the distributions. Spearman’s correlation test was performed between the Silver Index and instrumental gait parameters for each group (stroke, PD, and MS) at T0 and T2.

Values of *p* < 0.05 were considered significant. Statistical analysis was performed with SPSS 25 (IBM Corp., Armonk, NY, USA).

## 3. Results

As previously described [19,20], ninety patients were enrolled in the study: 30 patients with stroke, 30 with PD, and 30 with MS. Patients were randomized in a 1:1 ratio: the ARC Intellicare group consisted of 44 patients, while the paper-based group consisted of 46 patients. Thirteen patients dropped out of the study (the reasons are reported in the main study [19]). Table 1 shows the clinical and demographic characteristics of the patients included in the study.

Table 2A, on the other hand, shows the results of Spearman’s correlation between the risk of falling, assessed using the Silver Index, and the spatiotemporal parameters recorded using instrumental gait analysis.

Similarly, Table 2B shows the results of Spearman’s correlation between the risk of falling and kinematic parameters.

## 4. Discussion

This study analyzed the correlation between the risk of falling, calculated using the Silver Index by the hunova^®^ robotic platform, and the kinematic and spatiotemporal parameters of walking recorded through instrumental gait analysis.

In stroke patients, the correlation analysis between the Silver Index and spatiotemporal parameters at baseline showed moderately significant correlations for asymmetry (stance/swing phase, both affected side and unaffected side), double support (affected and unaffected side), gait cycle time (affected and unaffected side), and cadence. Similarly, at the end of the home-based rehabilitation treatment, the correlation values of stance and swing phase, double support, gait cycle time, and cadence ranged from moderate to strong. However, in patients with PD and MS, no statistically significant correlations emerged.

As for the kinematic parameters, no statistically significant correlations with the Silver Index emerged in any patient population.

This study found that the Silver Index, which assesses the risk of falling, showed significant correlations with various spatiotemporal gait parameters in post-stroke patients, but not in patients with PD and MS. In post-stroke patients, the correlations observed between the Silver Index and measures such as stance and swing asymmetry, increased double support, and reduced cadence are consistent with the literature that identifies asymmetry and altered step timing as sensitive markers of instability and fall risk. Gait asymmetry is considered a key indicator of biomechanical inefficiency and increased energy cost, with negative effects on dynamic stability after stroke [35]. It is also known that a higher percentage of double support represents a compensatory strategy to increase stability, typically associated with a higher risk of falling and reduced functional performance [36]. The strengthening of correlations at the end of treatment could be explained in two different ways: (i) rehabilitation treatment amplified individual differences in compensation strategy, or (ii) clinical change made the association between fall risk and spatiotemporal parameters more consistent because patients who improved showed simultaneous changes in both the Silver Index and gait parameters. Both explanations suggest that the Silver Index may be sensitive to changes in gait quality resulting from rehabilitation intervention.

Conversely, in subjects with PD and MS, the lack of significant correlations between the Silver Index and spatiotemporal or kinematic parameters can be interpreted by considering the pathophysiological characteristics specific to each disease. Assessing the risk of falls in older adults and neurological patients is a complex, multifactorial phenomenon involving postural control, locomotion, sensory integration, and reactive abilities [21]. The Silver Index is a composite index that integrates static and dynamic balance parameters, responses to perturbations, and stability limits, and provides an overall estimate of fall risk. In contrast, instrumental gait analysis primarily describes locomotion under controlled and repetitive conditions, often without environmental perturbations or reactive demands. Some authors have recently shown that the spatiotemporal and kinematic parameters of walking, although associated with the risk of falling, are not sufficient to represent all the domains of postural and motor control involved in actual falls [37]. In particular, the ability to recover balance, motor response times, and sensory integration is poorly correlated with gait metrics alone. The literature on posturography also highlights that center of pressure parameters only partially discriminate the risk of falling and depend on the experimental protocol [38]. In PD patients, this dissociation is further accentuated by the presence of the step quality: these are determinants of fall risk that are not fully captured by kinematic parameters or indices focused primarily on dynamic stability and temporal variability [7]. In PD, many falls are due to failures in protective postural responses (e.g., reactive stepping) and poor ability to quickly adapt stability in the presence of disturbances or changes in the environment, domains that are closer to what hunova^®^ assesses than what a standard gait analysis captures. It has also been shown that alterations in gait kinematics and postural instability may not be related in mild-to-moderate PD, highlighting that gait and balance deficits can progress in a partially independent manner [39]. A further element is task specificity: falls are often the result of an unexpected event (obstacle, turning, start/stop, external disturbance), while gait analysis may not include ecological conditions such as rapid turns, changes in direction, freezing triggers, or dual tasks, which are highly relevant in PD. The ability to improve or adapt stability during disturbed walking and compensatory responses may be altered in PD patients and may not emerge from simple spatiotemporal metrics [40]. Furthermore, the relationship between “how one walks” and “how much one falls” is mediated by fluctuating phenomena such as axial bradykinesia, trunk rigidity, postural instability, and impaired multisensory integration, which are recognized as components that are often refractory to treatment and cannot be reduced to single spatiotemporal or kinematic parameters [41]. The lack of correlation may also depend on the fact that many spatiotemporal parameters are strongly influenced by self-selected speed: the patient may voluntarily reduce speed as a precautionary strategy, apparently improving kinematic stability, but without correcting underlying reactive or sensory deficits (mechanisms on which the Silver Index weighs most heavily). Similarly, patients may “normalize” certain variables (e.g., stride length) through hip or trunk compensation, without regaining the ability to recover balance after a disturbance. The dissociation is accentuated by the role of freezing of gait (FOG) and ON/OFF fluctuations: many falls are closely related to episodes of freezing and motor state transitions, which may not occur during a single gait analysis acquisition or may appear intermittently. Some authors have highlighted how the contribution of FOG to falls is significant and dependent on circumstances and motor status, indicating that the dynamics of falls cannot be fully inferred from an “average” spatiotemporal profile [42]. Falls in PD are multifactorial in nature and include cognitive and behavioral components (attention, executive function, fear of falling, avoidance strategies), as well as environmental factors; these determinants are not measured by traditional gait analysis but can affect both the risk of falling and performance on balance tasks [43].

Similarly, the absence of statistically significant correlations between the risk of falling estimated using the hunova^®^ platform with the Silver Index and the spatiotemporal or kinematic parameters of gait analysis in MS patients is plausible because the two tools assess different functional domains. The Silver Index, in fact, mainly summarizes components of postural control and sensory integration (including dynamic and reactive conditions), which are markedly impaired in MS and are closely related to the probability of falling [44]. In contrast, gait analysis describes the locomotor pattern in often stable and predictable conditions, in which the patient can adopt cautious strategies (reducing speed, increasing double support) that “normalize” certain metrics without improving the reactive responses that are crucial for preventing falls. Furthermore, walking performance in the laboratory may not evoke the difficulties typical of real life (turns, obstacles, uneven surfaces), while dynamic balance tests with instruments and robotic platforms aim precisely to stimulate these mechanisms, showing clinical utility in MS [45]. In MS, gait impairment is highly multifactorial, and it is influenced by sensory and motor deficits, spasticity, muscle weakness, reduced dual-task adaptation capacity, fatigue, and vestibular problems, factors that may manifest intermittently and variably over time. A previous study showed that MS patients can adopt compensatory strategies during walking, such as reducing speed or increasing double support time, preserving apparently normal spatiotemporal parameters despite impaired postural control [44]. Evidence also indicates that the prediction of falls in MS improves when patient-reported and cognitive factors are included: for example, perceptions of dual-task difficulty and self-reported walking disability contribute to discriminating fallers [46]. Consistently, a systematic review has shown that dual-task tests are not always robust predictors of future fall events, suggesting that risk depends on multiple interactions between motor and cognitive domains that cannot be captured by single spatiotemporal variables [47]. Fear of falling, often fueled by fatigue, sleep disturbances, and depressive symptoms, is also a relevant and independent clinical determinant that can increase risk even in the absence of large observable kinematic variations [48]. Considering all this, a composite score such as the Silver Index, although sensitive to fall risk, may not be directly reflected in specific changes in range of motion or individual spatiotemporal measures in MS patients. The literature has shown that posturographic measures are strongly associated with the likelihood of falling in MS but show only weak or moderate correlations with gait analysis measures, suggesting a marked specificity of assessment tasks [49,50]. Furthermore, the risk of falling in MS is influenced by factors that are not purely biomechanical, such as cognitive deficits, reduced attention in dual-task conditions, fatigue, fear of falling, and elements that are not detected by standard instrumental gait analysis [51]. Reviews on MS and falls highlight the complexity and need for multifactorial measures (including questionnaires, cognitive measures, and fatigue) to predict the risk of falling in MS [52,53].

Under all conditions considered, no significant correlations emerged between the kinematic parameters and the Silver Index. This is consistent with the literature on global gait indices, which highlights how these synthetic measures more strongly reflect domains related to coordination, timing, and dynamic stability, rather than isolated joint range of motion alone [54]. Joint mobility, in fact, is a local measure that does not comprehensively describe the quality of postural control or the ability to compensate for disturbances, which are central elements in the genesis of fall risk.

Falls represent a serious healthcare issue, especially for people with neurological disorders. It has an impact on direct and indirect social costs, including the quality of life of patients and caregivers. Recurrent falls are linked to gait problems, which are common in middle and older adults, particularly in individuals with neurological diseases. Moreover, neurological gait impairments are more likely to cause falls than non-neurological problems [55]. This underlines the necessity of comprehensive gait assessments in identifying patients at increased fall risk. The assessment of risk of falls and its management and treatment is crucial [56,57]. Recent technological innovation, such as the hunova^®^ platform, could help professionals to estimate fall risk more accurately, in a non-invasive evaluation [20]. Based on this assessment and previous evidence, it is possible to prevent falls with an appropriate treatment [58,59], reducing the risk with a preventive and personalized approach [60] with a direct impact on the healthcare system.

The findings of the present study documented the progress made in comprehending and evaluating fall risk in neurological patients. The lack of correlation between the Silver Index and gait analysis parameters in patients with PD and MS does not indicate a lack of validity of the instruments, but rather that they measure different and complementary constructs. Therefore, a multimodal approach integrating reactive balance assessment, gait analysis, and cognitive factors appears more appropriate for estimating fall risk in patients with PD and MS. In this context, the absence of a correlation between the Silver Index and kinematic parameters is not a contradiction but rather confirms that these tools measure complementary aspects of motor function.

### Limitations of the Study

Despite the results obtained, this study has several limitations. First, it would be desirable to expand the sample size for each neurological population under study to confirm or refute the results obtained. It would also be appropriate to design an ad hoc study that considers the specificity of the pathology under study and long-term follow-up assessments to obtain prospective data. A more comprehensive understanding of fall risk and the identification of appropriate treatment may result from a multidisciplinary approach, including a cognitive dimension. To ensure consistency and comparability of results, future research should be focused on standardized techniques for evaluating falls across various neurological disorders.

## 5. Conclusions

The findings of the present ancillary study revealed an association between risk of falling and the spatiotemporal and kinematic gait parameters, and the need for a multidimensional approach in the evaluation and treatment of different neurological disorders. Overall, the results suggest that the Silver Index may be a useful tool for assessing post-stroke fall risk, as it is associated with biomechanical parameters well known for their role in gait stability. However, its applicability as an isolated measure appears limited in populations with PD and MS, for which fall risk assessment requires a multimodal approach encompassing cognitive, sensory, and functional aspects specific to the disease. It will therefore be necessary to integrate the use of the Silver Index with dedicated measures (e.g., freezing questionnaires for PD, fatigue scales, and dual-task assessments for MS) to obtain a more accurate characterization of fall risk.

## Figures and Tables

**Table 1:** Sample characteristics at baseline.

	ARC Intellicare Group	Paper-Based Group
**Whole sample**	n = 44	n = 46
Gender, n (%)		
Male	24 (54.55%)	24 (52.17%)
Female	20 (45.45%)	22 (47.83%)
Age (yr), mean ± SD	56.93 ± 13.01	55.15 ± 13.87
BMI, mean ± SD	24.66 ± 12.99	24.58 ± 14.04
Silver Index T0, mean ± SD	26.90 ± 17.90	28.20 ± 13.10
**Stroke**	n = 15	n = 15
Gender, n (%)		
Male	9 (60.00%)	11 (73.33%)
Female	6 (40.00%)	4 (26.67%)
Age (yr), mean ± SD	63.93 ± 7.87	64.33 ± 7.35
BMI, mean ± SD	23.62 ± 4.00	25.95 ± 3.58
Silver Index T0, mean ± SD	28.30 ± 12.20	29.40 ± 9.13
**Parkinson’s Disease**	n = 14	n = 16
Gender, n (%)		
Male	9 (64.29%)	8 (50.00%)
Female	5 (35.71%)	8 (50.00%)
Age (yr), mean ± SD	66.07 ± 5.66	61.93 ± 6.20
BMI, mean ± SD	25.00 ± 7.58	23.9 ± 2.50
Silver Index T0, mean ± SD	25.40 ± 15.60	24.10 ± 14.50
**Multiple Sclerosis**	n = 15	n = 15
Gender, n (%)		
Male	6 (40.00%)	5 (33.33%)
Female	9 (60.00%)	10 (66.67%)
Age (yr), mean ± SD	41.87 ± 6.69	38.73 ± 9.57
BMI, mean ± SD	22.4 ± 6.58	21.03 ± 6.45
Silver Index T0, mean ± SD	34.30 ± 23.90	30.60 ± 16.40

BMI: Body mass index.

**Table 2:** (**A**) Spearman’s correlation between Silver Index and spatiotemporal gait parameters for each neurological population at baseline (T0) and at the end of treatment (T2). (**B**) Pearson’s correlation between Silver Index and cinematic gait parameters for each neurological population at baseline (T0) and at the end of treatment (T2).

**(A)**						
	**T0**			**T2**		
	mean ± SD	r	*p*-value	mean ± SD	r	*p*-value
**Stroke**						
Stance phase AS (%)	62.07 ± 2.57	0.389	**0.007**	62.62 ± 3.27	−0.463	**0.017**
Stance phase US (%)	64.09 ± 4.33	0.333	**0.022**	64.00 ± 5.45	0.508	**0.008**
Swing phase AS (%)	37.93 ± 2.57	−0.389	**0.007**	37.38 ± 3.27	−0.463	**0.017**
Swing phase US (%)	35.91 ± 4.33	−0.333	**0.022**	36.00 ± 5.45	−0.508	**0.008**
Double support AS (%)	12.97 ± 2.47	0.446	**0.002**	13.33 ± 4.62	0.539	**0.005**
Double support US (%)	14.47 ± 8.59	0.270	0.066	13.35 ± 4.13	0.532	0.057
Gait cycle time AS (s)	1.34 ± 0.23	0.387	**0.007**	1.29 ± 0.42	0.560	**0.003**
Gait cycle time US (s)	1.35 ± 0.23	0.384	**0.008**	1.29 ± 0.43	0.557	**0.003**
Cadence (step/min)	91.73 ± 13.90	−0.327	**0.025**	94.27 ± 15.57	−0.553	**0.003**
Step length AS (mm)	0.45 ± 0.10	−0.106	0.477	0.47 ± 0.10	−0.207	0.309
Step length US (mm)	0.46 ± 0.10	−0.256	0.083	0.48 ± 0.11	−0.438	0.065
Mean velocity AS (m/s)	1.81 ± 0.50	−0.206	0.185	1.95 ± 0.53	−0.463	0.187
Mean velocity US (m/s)	1.87 ± 0.44	−0.246	0.096	2.01 ± 0.50	−0.455	0.059
Gait cycle length AS (mm)	0.98 ± 0.22	−0.199	0.179	1.03 ± 0.23	−0.334	0.096
Gait cycle length US (mm)	0.98 ± 0.22	−0.203	0.171	1.03 ± 0.23	−0.334	0.095
Step width AS (mm)	0.16 ± 0.03	0.117	0.435	0.17 ± 0.02	0.149	0.466
Step width US (mm)	0.16 ± 0.03	0.117	0.435	0.17 ± 0.02	0.149	0.466
**Parkinson’s Disease**						
Stance phase AS (%)	60.54 ± 2.76	−0.125	0.518	59.80 ± 2.53	0.100	0.634
Stance phase US (%)	60.80 ± 2.57	0.027	0.888	59.99 ± 2.30	0.039	0.854
Swing phase AS (%)	39.46 ± 2.76	0.125	0.518	40.20 ± 2.53	−0.100	0.634
Swing phase US (%)	39.20 ± 2.56	−0.027	0.888	40.01 ± 2.30	−0.039	0.854
Double support AS (%)	10.85 ± 2.68	−0.120	0.534	9.87 ± 2.39	0.108	0.607
Double support US (%)	10.66 ± 2.55	0.015	0.940	10.03 ± 2.44	0.095	0.653
Gait cycle time AS (s)	1.14 ± 0.10	−0.148	0.443	1.11 ± 0.10	−0.091	0.667
Gait cycle time US (s)	1.14 ± 0.11	−0.151	0.435	1.11 ± 0.10	−0.080	0.705
Cadence (step/min)	105.89 ± 9.52	0.114	0.557	109.33 ± 9.91	0.094	0.655
Step length AS (mm)	0.53 ± 0.07	0.166	0.388	0.55 ± 0.05	0.167	0.426
Step length US (mm)	0.53 ± 0.07	0.025	0.898	0.55 ± 0.06	−0.071	0.735
Mean velocity AS (m/s)	2.32 ± 0.35	0.098	0.614	2.45 ± 0.35	0.252	0.225
Mean velocity US (m/s)	2.32 ± 0.35	0.115	0.553	2.46 ± 0.37	0.127	0.546
Gait cycle length AS (mm)	1.14 ± 0.15	0.136	0.481	1.20 ± 0.13	0.057	0.788
Gait cycle length US (mm)	1.14 ± 0.15	0.152	0.431	1.19 ± 0.13	0.072	0.734
Step width AS (mm)	0.15 ± 0.03	−0.091	0.637	0.15 ± 0.02	0.049	0.817
Step width US (mm)	0.15 ± 0.03	−0.091	0.637	0.15 ± 0.02	0.049	0.817
**Multiple Sclerosis**						
Stance phase AS (%)	63.58 ± 4.08	−0.360	0.056	63.05 ± 4.39	0.073	0.728
Stance phase US (%)	63.43 ± 4.68	0.041	0.832	62.84 ± 4.28	0.162	0.440
Swing phase AS (%)	36.42 ± 4.08	0.360	0.056	36.95 ± 4.39	−0.073	0.728
Swing phase US (%)	36.57 ± 4.68	−0.041	0.832	37.16 ± 4.28	−0.162	0.440
Double support AS (%)	13.18 ±3.41	−0.209	0.267	13.14 ± 3.65	0.076	0.719
Double support US (%)	13.95 ± 4.20	−0.223	0.237	13.30 ± 3.61	0.159	0.448
Gait cycle time AS (s)	1.30 ± 0.25	−0.020	0.918	1.26 ± 0.18	−0.050	0.812
Gait cycle time US (s)	1.31 ± 0.28	−0.045	0.812	1.26 ± 0.18	0.020	0.926
Cadence (step/min)	93.01 ± 17.45	0.111	0.558	97.13 ± 12.35	0.009	0.965
Step length AS (mm)	0.50 ± 0.09	0.208	0.271	0.52 ± 0.08	0.140	0.504
Step length US (mm)	0.50 ± 0.09	0.247	0.189	0.52 ± 0.08	0.104	0.620
Mean velocity AS (m/s)	2.14 ± 0.45	0.271	0.147	2.19 ± 0.57	0.039	0.852
Mean velocity US (m/s)	2.12 ± 0.37	0.319	0.086	2.18 ± 0051	0.048	0.818
Gait cycle length AS (mm)	1.10 ± 0.18	0.361	0.056	1.12 ± 0.17	0.161	0.441
Gait cycle length US (mm)	1.09 ± 0.17	0.326	0.079	1.12 ± 0.17	0.142	0.497
Step width AS (mm)	0.16 ± 0.04	0.129	0.498	0.16 ± 0.03	0.069	0.742
Step width US (mm)	0.16 ± 0.04	0.129	0.498	0.16 ± 0.03	0.054	0.799
(**B**)						
	**T0**			**T2**		
	mean ± SD	r	*p*-value	mean ± SD	r	*p*-value
**Stroke**						
Hip ROM AS (deg)	38.70 ± 7.11	−0.214	0.149	38.74 ± 6.53	−0.249	0.220
Hip ROM US (deg)	40.53 ± 6.24	−0.219	0.138	40.41 ± 6.09	−0.223	0.273
Knee ROM AS (deg)	49.43 ± 9.19	−0.096	0.519	51.15 ± 7.26	−0.201	0.325
Knee ROM US (deg)	52.53 ± 7.84	−0.014	0.926	53.06 ± 7.20	0.037	0.859
Ankle ROM AS (deg)	20.73 ± 4.74	−0.094	0.528	21.17 ± 3.81	−0.376	0.058
Ankle ROM US (deg)	22.79 ± 5.70	−0.096	0.519	24.54 ± 5.13	−0.134	0.513
**Parkinson’s Disease**						
Hip ROM AS (deg)	44.10 ± 6.02	0.331	0.080	43.85 ± 6.43	0.134	0.523
Hip ROM US (deg)	45.36 ± 9.65	−0.143	0.460	44.56 ± 5.48	0.142	0.500
Knee ROM AS (deg)	49.15 ± 11.75	−0.098	0.613	51.00 ± 9.86	−0.243	0.241
Knee ROM US (deg)	48.17 ± 11.69	−0.237	0.216	50.13 ± 10.89	−0.304	0.140
Ankle ROM AS (deg)	29.63 ± 9.24	−0.054	0.780	28.91 ± 7.61	−0.014	0.948
Ankle ROM US (deg)	33.80 ± 12.45	0.219	0.253	32.51 ± 10.82	−0.085	0.686
**Multiple Sclerosis**						
Hip ROM AS (deg)	45.83 ± 8.15	0.274	0.143	44.84 ± 6.32	0.118	0.575
Hip ROM US (deg)	43.91 ± 8.30	0.189	0.317	44.56 ± 8.13	0.252	0.225
Knee ROM AS (deg)	45.90 ± 13.88	0.392	0.056	48.16 ± 11.29	−0.042	0.844
Knee ROM US (deg)	45.47 ± 13.04	0.243	0.197	48.87 ± 10.99	−0.093	0.658
Ankle ROM AS (deg)	29.72 ± 12.31	0.066	0.729	30.94 ± 12.68	0.130	0.536
Ankle ROM US (deg)	29.06 ± 12.77	0.4204	0.055	27.88 ± 11.05	−0.092	0.661

AS: affected side; US: unaffected side; SD: standard deviation; r: Spearman’s correlation test value; *p*-value: probability value; s: seconds; min: minutes; m: meters; mm: millimeters. r values: 0.2–0.3 weak correlation; 0.3–0.5 moderate correlation; above 0.5 high correlation. In bold significant values for *p* < 0.05.

## Data Availability

The raw data supporting the conclusions of this article will be made available by the authors on request.

## Supplementary Materials

The following supporting information can be downloaded at: https://www.mdpi.com/article/10.3390/s26030840/s1. Table S1. Gait Analysis Parameters.
