# Supplementary material for: Assessment of Fall Risk in Neurological Disorders and Technology: Relationship Between Silver Index and Gait Analysis
Source: Sensors (Basel). 2026 Jan 27;26(3):840. doi: 10.3390/s26030840 (PMC12899951; doi:10.3390/s26030840)
Supplement: Supplementary file 1 [file sensors-26-00840-s001.zip › sensors-4043077-supplementary.pdf]

**Table S1.** Gait Analysis Parameters

| Variables | Definition / Measurements (units) |
|---|---|
| **Spatial-Temporal** | |
| Stance phase | Time spent during the stance phase (% of gait cycle) |
| Swing phase | Time spent during the swing phase (% of gait cycle) |
| Double support | Time spent on both feet during the gait cycle (% of gait cycle) |
| Gait cycle time | Seconds |
| Cadence | Steps per minute |
| Step length | Distance between 2 subsequent contralateral heel strikes (millimeters) |
| Mean velocity | Distance covered per unit of time (meters/seconds) |
| Gait cycle length | Millimeters |
| Step width | Distance between the midpoint of the 2 heels (millimeters) |
| **Cinematic** | |
| Hip ROM | Maximal range of excursion of the hip (degrees) |
| Knee ROM | Maximal range of excursion of the knee (degrees) |
| Ankle ROM | Maximal range of excursion of the ankle (degrees) |

*ROM: Range of Motion*
